# Chronotypology and melatonin alterations in minimal hepatic encephalopathy

**DOI:** 10.1186/1740-3391-7-6

**Published:** 2009-05-29

**Authors:** Dimitrios Velissaris, Vasilis Karamouzos, Panagiotis Polychronopoulos, Menelaos Karanikolas

**Affiliations:** 1 Department of Anaesthesiology and Critical Care Medicine, Patras University Hospital, Rion 26500, Greece; 2 Department of Neurology, Patras University Hospital, Rion 26500, Greece

## Abstract

**Background:**

"Minimal (subclinical) hepatic encephalopathy" is a term that describes impairment of every day life activities in cirrhosis patients without clinical neurologic abnormalities. Melatonin diurnal pattern disruption and metabolic changes due to liver insufficiency can affect the human biologic clock. Our study was conducted to measure plasma melatonin levels in an attempt to correlate plasma melatonin abnormalities with liver insufficiency severity, and describe chronotypology in cirrhosis patients with minimal encephalopathy.

**Methods:**

Twenty-six cirrhotic patients enrolled in the study and thirteen patients without liver or central nervous system disease served as controls. All patients had full clinical and biochemical evaluation, chronotypology analysis, neurological evaluation, melatonin profile and quality of life assessment.

**Results:**

Cirrhotic patients with minimal encephalopathy exhibit melatonin secretion abnormalities. Cirrhosis patients with more severe hepatic insufficiency (Child-Pugh score > 5) had significantly (p < 0.04) lower evening melatonin levels compared to patients with less severe insufficiency (Child-Pugh score = 5).

Chronotypology analysis revealed Morning Type pattern in 88% of cirrhosis patients.

**Discussion:**

The presence of abnormal plasma melatonin levels before the onset of clinical hepatic encephalopathy, and the finding that patients with more severe cirrhosis have lower evening melatonin levels are the most important findings of this study. Despite these melatonin abnormalities, chronotypology revealed Morning Type pattern in 23 of 26 cirrhosis patients. We believe these findings are important and deserve further study.

**Conclusion:**

Melatonin abnormalities occur in cirrhosis patients without clinical encephalopathy, are related to liver insufficiency severity, may influence chronotypology patterns, and certainly deserve further investigation.

## Background

Most physiological and behavioral human variables, including sleep and wakefulness, endocrine function, thermoregulation and metabolism exhibit circadian patterns. Circadian rhythms are controlled by central neural pacemakers, of which the Suprachiasmatic Hypothalamic Nucleus (SCN) is the best characterized [1-5] Hepatic encephalopathy, a major cirrhosis complication, is a clinical syndrome characterized by mental status abnormalities in patients with severe hepatic failure. In contrast, the term "minimal (subclinical) hepatic encephalopathy" describes milder disturbances of biological parameters such as sleep, and abnormalities in every day life activities, in the absence of clinical encephalopathy. [6-12] Liver diseases are often associated with hormonal disorders, and metabolic changes in cirrhosis can result in circadian rhythm abnormalities. Previous studies have shown that disruption of the diurnal rhythm of melatonin reflects circadian function alterations that contribute to the disturbances of the sleep-wake cycle frequently seen in patients with cirrhosis. [13-15]. This study was designed to evaluate the melatonin profile of cirrhotic patients and correlate melatonin abnormalities to cirrhosis severity in the absence of clinical hepatic encephalopathy.

## Methods

### Patients

Twenty-six cirrhotic patients (20 men, 6 women) enrolled in the study. Mean patient age was 65.1 ± 10.7 years in men and 62.8 ± 3.4 years in women. Cirrhosis etiology was alcohol in 13 patients, HBV infection in 9, HCV infection in 1, combined alcohol and HBV infection in 1 and combined alcohol and HCV infection in 1. Cirrhosis etiology was unknown in one case. The diagnosis of cirrhosis was confirmed by liver biopsy in all patients. All patients had regular follow-up visits in our Liver Outpatient Clinic. Patients receiving medications with Central Nervous System (CNS) effects were excluded.

All patients underwent comprehensive clinical and biochemical evaluation. Twenty-two cirrhosis patients were Child-Pugh class A (16 had score 5 and 6 had score 6), and 4 patients were Child-Pugh class B (1 score 7, 2 score 8 and 1 subject with score 9).

Thirteen patients hospitalized for chronic diseases (5 with COPD, 4 with lung cancer, and 4 with autoimmune bowel disease) but without liver or CNS involvement were included in the study as controls. Mean control age was 67.8 ± 10.8 years.

Psychometric tests, Chronotypology and Neurologic Assessment, as described below, were performed in all cirrhosis and control patients. The research protocol was approved by the Institution Ethics Committee, and written informed consent was obtained from all patients before entering the study.

### Psychometric Tests

The psychometric status of cirrhosis patients was evaluated with the Number Connecting Test A (NCT-A), the Digit Symbol Test (DST) and the Sickness Impact Profile (SIP) [16,17]. The NCT-A measures cognitive motor activity and the DST measures motor speed and accuracy. Daily functioning was measured with the SIP, a quality of life questionnaire containing several items on daily function.

### Sleep History-Chronotypology

Sleep history was assessed with:

a) A self-assessment questionnaire to determine morningness vs. eveningness chronotypology according to Horne-Ostberg analysis [18]. This test includes 19 questions concerning usual habits, social and personal actions, and day-night behavior and calculates a score, thereby assigning a characteristic morning-evening type to each patient. This score can result in 5 different chronotypology types, described as Definitely Morning (DM), Moderately Morning (MM), Neither Type (NT), Moderately Evening (ME), and Definitely Evening (DE).

b) The Basic Nordic Sleep Questionnaire (BNSQ), which consists of 21 standardized questions, including 27 items on sleep and sleep disorders [19].

Both tests were translated in Greek, with questions adapted to the social habits and particular characteristics of the Greek population.

### Neurologic assessment-EEG

All patients underwent a comprehensive physical and neurophysiologic neurologic examination with emphasis on cortical function assessment. An awake 16-channel digital EEG was used. Abnormal EEG findings were classified as specific (epileptiform or paroxysmal) or non-specific (theta and delta waves in various combinations) disturbances. Non-specific disturbances were further classified as mild, moderate or severe.

### Hormones

Blood samples for hormone assays were collected in the morning (09.00), midday (14.00) and evening (21.00). Melatonin levels were measured with the Radioimmunoassay method by Biosourse (8 rue de I: Industrie-B-1400 Nivelles, Catalog Nr. KIPLO800).

### Statistical Analysis

Data normality was assessed with the Kolmogorov-Smirnov test. Normally distributed descriptive data are presented as mean ± SD. Differences between groups were compared with ANOVA or with the Student's t test, as appropriate. Values at different time points within each group were compared with repeated measures ANOVA. The Mann-Whitney test was used for data that were not normally distributed. All data analysis was done with the SPSS v15 Statistical Software package (SPSS, Chicago, IL) on a Windows-based PC.

## Results

### Liver Function and Neurological Evaluation

Clinical and Laboratory results used to calculate Child Scores in cirrhosis patients and in controls are presented in Table 1.

**Table 1:** Liver function and clinical data used in calculating Child Scores

**Parameter**	**Cirrhosis**	**Controls**
Bilirubin (total, mg/dL)	1.44 ± 0.95	0.89 ± 0.24
Albumin (gm/dL)	4.0 ± 0.5	3.7 ± 0.9
PT (secs)	14.4 ± 1.8	11.4 ± 0.8
Ascites	2/26	0/13
Enceplalopathy	0/26	0/13

Data are presented as proportion or as mean ± SD

Detailed neurologic physical examination showed normal muscle tone, normal tendon reflexes and absence of flapping tremor in all cirrhosis patients. These findings demonstrate minimal hepatic insufficiency, absence of biochemically active liver disease and absence of clinical hepatic encephalopathy in our cirrhosis population. There were no neurologic disturbances in the control group.

### Psychometric Tests

The NCT-A and DST tests showed that our cirrhotic subjects had impaired psychomotor speed and attention performance, with longer calculated time (> 90 sec) compared to what is described in the literature for normal subjects. In addition, cirrhotics had diminished daily functioning level, as reflected by significant impairment in all SIP categories. Cirrhosis patients took longer (> 20 minutes) to complete the SIP test, as compared to less than 20 minutes in healthy individuals. These findings confirm the presence of minimal hepatic encephalopathy in our population. There were no abnormalities in any of the above tests in the control group.

#### Sleep history-Chronotypology

Quantitative sleep history analysis with the Horne score showed that 2/26 cirrhotic patients (7.7%) were DM, 21/26 patients (80.8%) were MM, and 3/26 (11.5%) had no particular day-night behavior characteristics (NT). Qualitative sleep analysis with the BNSQ test showed that sleep history was abnormal in 24/26 cirrhotic patients (92.3%). Observed sleep pattern abnormalities included prolonged sleep latency (> 30 minutes), short (< 6 hours) duration of night-sleep and frequent awakenings (> 3/night). These sleep abnormalities were not present in the control group. The Horne score showed that all patients in the control group had MM chronotypology.

### EEG

EEG was performed in 22 of 26 cirrhosis patients and 13 of 13 controls. Non-specific EEG disturbances were present in 11 of 22 patients (50%). These disturbances consisted of theta or delta waves, and were graded as mild (7 patients), moderate (3 patients), or severe (1 patient). We did not find epileptiform discharges in any cirrhosis patient. There were no EEG disturbances in the control group.

### Melatonin diurnal variation

Melatonin concentration measurements in cirrhosis patients and in controls are summarized in Table 2. Melatonin levels in cirrhosis were significantly different between 09.00 and 14.00 (p < 0.05) and between 09.00 and 21.00 (p < 0.01).

**Table 2:** Plasma Melatonin levels (pg/ml) in patients with cirrhosis and in controls

Time	Cirrhosis	Controls	P
09.00	5.77 ± 7.74	3.40 ± 2.71	NS
14.00	2.60 ± 2.37	2.53 ± 1.43	NS
21.00	1.55 ± 1.29	1.64 ± 0.73	NS

Results presented as mean ± SD. NS means "Non-Significant".

**Figure 1:**
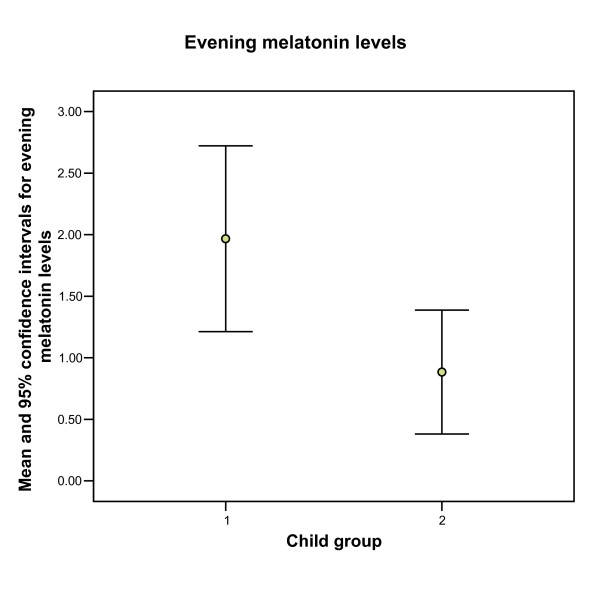
**Evening melatonin levels and cirrhosis severity**. Patients with Child score > 5 (group 2) have significantly (p < 0.04) lower evening melatonin levels compared to cirrhosis patients with Child score ≤ 5 (group 1)

In order to assess the association between hormone measurements and the degree of liver failure according to Child-Pugh score, we divided cirrhosis patients in two groups: those with Child-Pugh score = 5 (n = 16) and those with score > 5 (n = 10). Figure 1 shows that cirrhosis patients with more severe hepatic insufficiency (Child-Pugh score > 5) had significantly (p < 0.04) diminished evening melatonin levels compared to those with less severe insufficiency (Child-Pugh score = 5).

## Discussion

Abnormal circadian rhythms have been described for several biological parameters in cirrhosis. The existence of a "biologic clock" in the SCN allows the body to anticipate external environment modifications during the day-night cycle. Current views propose two explanations for circadian alterations seen in chronic liver disease. According to the first hypothesis, circadian rhythm abnormalities arise from the effect of neurotoxins on the SCN and/or its afferent/efferent connections. According to the second hypothesis, impaired hepatic melatonin clearance, probably due to decreased liver blood flow, lower 6-beta-hydroxylase activity, and competition with bilirubin in the intrahepatic transport system [14,20-22], results in elevated morning melatonin levels, thereby causing a circadian clock phase-shift. It is possible that both proposed mechanisms, by combining the effects of hepatocellular dysfunction and portal systemic shunting, are responsible for circadian abnormalities in liver disease [1,23-25].

Melatonin has been proposed to act as "internal synchronizer" for circadian rhythms generated at different levels of the human body. Melatonin alterations have been described in many biological rhythm disorders, including sleep disorders related to jet lag, shift work, blindness and aging [13,15,26-30]. Cirrhosis patients have markedly elevated daytime melatonin levels, significantly delayed melatonin increase onset and consistently delayed melatonin peak [14,20-22]. High daytime melatonin levels cause an endogenous biologic clock phase-shift; this diurnal melatonin disruption probably has clinical implications and may be related to the high prevalence of sleep disturbances [7,31] and pituitary hormone abnormalities [32] in cirrhosis. Hyperammonemia, cerebral accumulation of false neurotransmitters, abnormal dopaminergic activity, GABA-ergic neurotransmission disturbances, and stress are additional factors possibly affecting the biologic clock in cirrhosis.

Sleep disturbances, such as delayed sleep onset and short night sleep duration, which are well documented in cirrhosis, were also observed in our minimal encephalopathy cirrhosis population. Cirrhosis patients generally exhibit sleep-wake cycle abnormalities, manifesting as chronotypology other than Morningness Type. However, in striking disagreement with previous findings [13,14,24], 23 of 26 (88.4%) of our cirrhosis patients exhibited Morningness (DM or MM) Chronotypology. This unexpected finding may in part be explained by the opposing effects of melatonin and bright morning light in our Mediterranean population. Although other factors may also affect chronotypology, we believe that the true importance of melatonin deserves further investigation.

Limitations of this study include study design (observational study, no intervention, no randomization, no power analysis), the small number of patients both in the cirrhosis and in the control group, and the fact that we have tried to make inferences about melatonin secretion from only three measurements per day.

The most important finding of our study is the presence of abnormal plasma melatonin levels in cirrhosis patients before the onset of clinical hepatic encephalopathy. This is not an entirely new finding, as it has also been reported by Steindl et al [14]. However, the study by Steindl, which only included 7 cirrhosis patients, compared cirrhosis patients with subclinical encephalopathy vs. healthy controls. In contrast, our study included 26 cirrhosis patients and 13 controls, and we used patients with other diseases, rather than healthy people, as controls. Compared to the Steindl study [14] our study shows two additional important findings: i) the observation that evening melatonin levels are lower in patients with more severe cirrhosis (Figure 1) and ii) the observed morning type chronotypology in our cirrhosis population.

These findings are important because they are highly abnormal and markedly different compared to values reported in the literature for normal subjects. The finding that patients with more severe cirrhosis have lower evening melatonin levels has not, to our knowledge, been described before. In addition, the observed chronotypology raises questions regarding the influence of melatonin on sleep patterns in cirrhosis.

Further studies are needed to accurately describe melatonin secretion and clarify the possible association between melatonin levels and chronotypology in cirrhosis.

## Conclusion

Melatonin abnormalities occur in patients with liver cirrhosis in the absence of clinical encephalopathy and are related to severity of cirrhosis. Morning type chronotypology was identified in our cirrhosis patients. The influence of these melatonin abnormalities on chronotypology patterns in cirrhosis patients with subclinical encephalopathy deserves further investigation.

## Abbreviations

HBV: Hepatitis B Virus; HCV: Hepatitis C Virus; DM: Definitely Morning Type; MM: Moderately Morning Type; NT: Neither Type; ME: Moderately Evening Type; DE: Definitely Evening Type; BNSQ: The Basic Nordic Sleep Questionnaire; NCT-A: Number Connecting Test A; DST: Digit Symbol Test; SIP: Sickness Impact Profile; SD: Standard Deviation; ANOVA: Analysis Of Variance.

## Competing interests

The authors declare that they have no competing interests.

## Authors' contributions

DV is the principal investigator, participated in the design of the study and performed research. VK helped with data collection, paper editing and submission. PP did all neurologic and chronotypology evaluations. MK performed the statistical analysis, interpreted results and wrote the paper. All authors read and approved the final manuscript.
